# COVID-19: A Window of Opportunity for Positive Healthcare Reforms

**DOI:** 10.34172/ijhpm.2020.66

**Published:** 2020-05-02

**Authors:** Stefan Auener, Danielle Kroon, Erik Wackers, Simone van Dulmen, Patrick Jeurissen

**Affiliations:** Radboud University Medical Center, Radboud Institute for Health Sciences, IQ Healthcare, Nijmegen, The Netherlands.

**Keywords:** COVID-19, Healthcare Reform, E-Health, Low-Value Care, Health Policy

## Abstract

The current coronavirus disease 2019 (COVID-19) pandemic is testing healthcare systems like never before and all efforts are now being put into controlling the COVID-19 crisis. We witness increasing morbidity, delivery systems that sometimes are on the brink of collapse, and some shameless rent seeking. However, besides all the challenges, there are also possibilities that are opening up. In this perspective, we focus on lessons from COVID-19 to increase the sustainability of health systems. If we catch the opportunities, the crisis might very well be a policy window for positive reforms. We describe the positive opportunities that the COVID-19 crisis has opened to reduce the sources of waste for our health systems: failures of care delivery, failures of care coordination, overtreatment or low-value care, administrative complexity, pricing failures and fraud and abuse. We argue that current events can canalize some very needy reforms to make our systems more sustainable. As always, political policy windows are temporarily open, and so swift action is needed, otherwise the opportunity will pass and the vested interests will come back to pursue their own agendas. Professionals can play a key role in this as well.

## Background

The current coronavirus disease 2019 (COVID-19) pandemic is testing healthcare systems like never before. Italy (2) and Spain (7), both close to the top of the World Health Organization (WHO) ranking of best health systems,^1^ are struggling with many deaths and overcrowded hospitals. The United States has surpassed these countries with victim estimates that might exceed 80 000.

All efforts are now being put into controlling the COVID-19 crisis. With an emerging economic recession, and a country such as Italy carry debt well above 100% of gross domestic product, this implies severe fiscal pressure for health systems, once the current health crisis is over. However, we also witness many positive things happening. Institutions and regulations have become very flexible. Decisions that otherwise would have taken months of deliberation are now made instantly. At the same time, some ad hoc policy changes are taken without a solid foundation aimed at reducing threats and might also offer opportunities for actors to exert political power and pursue personal interest. On the other hand, we can and should harvest the possibilities that are opening up. For this, we among other things need policy entrepreneurs that can pursue and steer the current debate towards a sustainable transformation of healthcare delivery.

In this perspective we analyze these ‘forces-for-the-good’ and propose measures how to include them structurally in our health systems. We focus on lessons from COVID-19 to increase the sustainability of health systems and to prepare for follow-up policies of austerity. If we catch the opportunities, the crisis might very well be a policy window for positive reforms.

Berwick and Hackbarth have pointed towards six domains as ‘sources of waste.’^2^ COVID-19 presents a window of opportunity to tackle such ‘sources of waste’ (Table): (1) *Failures of care delivery:* Telehealth and digitalization are suddenly being used like never before; public health is back in the center of attention; scalability of vital functions is important. (2) *Failures of care coordination:* We have witnessed a lot of centralization of vital functions of healthcare; the importance of readily available integral data has never been more clear. (3) *Overtreatment or low-value care:* The amount of low-value care diminishes drastically. (4) *Administrative complexity:* COVID-19 illustrates how regulatory barriers have suddenly become lean, while some new regulatory barriers are established to control the current and prevent new outbreaks. (5) *Pricing failures:* Wired cross-subsidies between the different parts of the healthcare system have become visible. (6) *Fraud and abuse:* Finally, and as result of huge flows of resources towards healthcare, inevitably some possibilities will open up for charging extreme prices or otherwise collecting public means. The remainder of this perspective, illustrates the most positive opportunities that the COVID-19 crisis has opened to reduce the sources of waste for our health systems.

**Table:** Sources of Waste and Opportunities for Reform

**Berwick and Hackbarth’s Definition** ^2^	**Domain Components** ^3^	**Current Events**	**Advices for Reform**
Failures of care delivery *‘Poor execution or lack of widespread adoption of known best care processes.’*	Patient safety Preventive care Practice- and delivery system-based inefficiency	Cost-effectiveness of public health made clear Postponing elective high value care E-health widely adopted Scalability of acute care functions Acute care out crowds other delivery functions	Strengthen international governance and the monitoring and detecting functions of diseases Prioritizing high value care Make current e-health levels the new normal Rigorous real world effectiveness evaluations Create spare capacities necessary supplies and (human) capital Redirecting the delivery system towards separate workstreams
Failures of care Coordination *‘Patients fall through the slats in fragmented care.’*	Reduce unnecessary hospitalizations Coordination super utilizers/complex patients Seamless transitions of care Integrated data systems	Coordination of acute care centralized Higher COVID-19 mortality Quick transmissions between care levers Timely available data of key importance	Align governance with specific subsystems Design structures around co-morbidity as the new normal Create central integrated data system Create central integrated data system
Overtreatment or low-value care *‘Subjecting patients to care that, according to sound science and the patient’s own preferences, cannot possibly help them.’*	Low-value care Overdiagnosis Overprescribing Overuse in end of life care	Low-value care comes to a hold Large scale data show treatment differentials as a result of the COVID shock	Top-down approach to prevent a V-shape recurrence of low value care Measurement of watchful waiting situation that has arisen Stricter purchasing according to type of care Lower reimbursement elective/chronic care
Administrative complexity *‘Government, accreditation agencies, payers, and others create inefficient or misguided rules.’*	Inefficient or misguided rules Billing and coding	Immediate needs prevail regulations	Put a (pseudo)price on administrative complexities by payers
Pricing failures *‘Prices migrate far from those expected in well-functioning markets.’*	Absence of effective transparency and competitive markets	Inherent cross-subsidies towards acute care become visible	Ending cross subsidies
Fraud and abuse *‘The waste that comes as fraudsters issue fake bills and run scams.’*	Costs of fraud and abuse	The need for essential goods (ventilators, masks) leads to extreme pricing	Coordinate purchasing of vital goods on the global market

Abbreviation: COVID-19, coronavirus disease 2019.

## Reducing Failures in Health Delivery: Telehealth and Protecting Public Health

### Telehealth and Digital Care

Despite all the challenges, the COVID-19 crisis may be a huge opportunity for technologies that overcome space and distance. During this crisis, we have seen quick responses towards providing guidelines concerning the virus and social distancing. Several online self-tests provide triage possibilities, thereby reducing physician workload. Furthermore, telemonitoring applications for suspected corona patients are currently developed and adopted. These applications allow us to monitor symptoms and deterioration. Collection of (anonymized) data allows for tracking and prediction of infection rates, which allows for more flexibility in policy responses to future outbreaks.

On an immediate note, studies have shown that lockdowns have major impacts on mental health^4^ and technology might mitigate some of these adverse effects right now but also in the foreseeable future. Digital applications such as e-coaches may prove valuable or even critical to reduce this scarcity by substitution or enabling mental health workers to overcome physical distance.^5^

However, maybe most notably is an acceleration in the adoption of existing telemonitoring initiatives. For example, a primary care office in the province of North Brabant, the hardest hit region in the Netherlands, showed a sharp decline in general practice visits while simultaneously witnessing a doubling of e-consultations and consultations by phone^6^ (see Figure). The same holds for non COVID-19 consultations by physicians elsewhere in the country.

**Figure:**
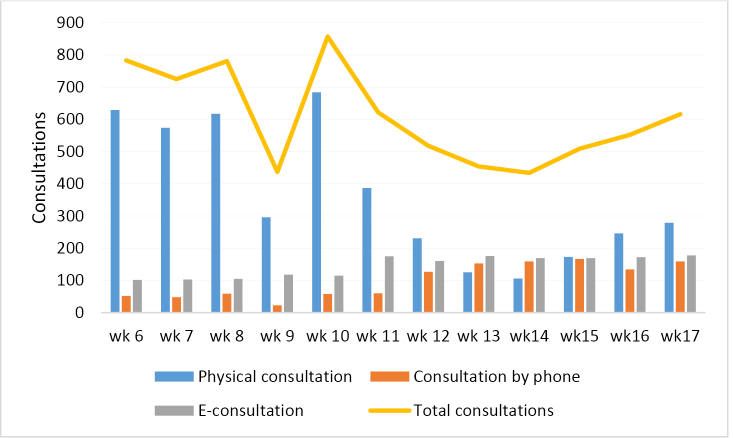
Increase in E-Consultations and Consultations by Phone in a General Practice.

What otherwise might have taken more than a year is due to the COVID-19 crisis now being done instantly. The Maastricht university medical center in the Netherlands made telemonitoring available to all patients with chronic heart failure.^7^ Teladoc, a company focused on telemonitoring, witnessed an increase of 50% for daily virtual medical visits in the second week of March.^8^

Telehealth seems to suddenly have matured and this holds great prospects to keep our health systems sustainable. The newly created familiarity with video calling may result in both patients and physicians being more open to use these technologies. The COVID-19 crisis unintendedly resulted in one of the largest trials of e-health within regular care paths for a variety of diseases. Governments, healthcare insurers, and healthcare providers should evaluate the effects of this transition and use the learning curve and experiences from healthcare providers that quickly adopted e-health to support other healthcare providers.

### Protecting Public Health

The Organisation for Economic Co-operation and Development (OECD) pointed the high cost-effectiveness of antimicrobial resistance interventions.^9^ COVID-19 easily dwarfs these very positive ratios. More than any mathematical model, COVID-19 illustrates how the cost-effectiveness of classical public health protection policies surpasses even the most optimistic calculations. The crisis underlines the need to strengthen our basic infrastructure on threats towards public health: international governance, monitoring and detecting (testing, testing, testing) functions of diseases should be improved. With curative therapies still lacking, questions such as how to make people comply with social distancing do pop up. The need of mature behavioral public health comes to the fore, together with readily available data. Finally, we need to fix current failures in developing new therapies for global health threats, such as antibiotics. Pharmaceutical manufacturers should be confronted with much stronger incentives to tackle these needs.

## Reducing Overtreatment or Low-Value Care

Healthcare providers currently focus on increasing capacity for and treating critical ill patients. This leads to postponing diagnostics and elective care.^10^ This includes necessary high-value care, such as chemotherapy and cardiac surgery. The degree to which such forced delays lead to actual harm in people’s health, and involve additional cost, has yet to be researched. It should be prevented by restarting this care as soon as possible. To prevent even more harm, high-value and urgent necessary care should be prioritized over low-value care. Along with this prioritizing of care, dilemmas arise on the value of hospital care and intensive care unit (ICU) care for patients with short-life expectancy (including strategies of palliative care), and the consequent oppression of valuable care for other patients.

COVID-19 probably will over the short term keep stimulating rigorous prioritization of acute and high-value patient care over low-value care. Postponing the majority of the elective diagnostics and treatments shifts the approach from acting now to watchful waiting. If this is adequately monitored, it could provide us with better real-life data on the value of care. This data hands new options to purchasers and policy-makers to prevent the restart of low-value care. For example by adjusting their strategies and purchase less volume of certain care interventions (ending fee-for-service mechanisms). It can also guide discussions with scientific organizations and hospitals on priorities for healthcare delivery in the future.

An estimated $75.7 billion to $101.2 billion is spent annually on overtreatment or low-value care in the United States alone. Most other countries will not be that far off when converted to the ratio of total spending. With upcoming budgetary difficulties, this could be the time to prevent costs of underuse on high-value care, and tackle low-value care by restricting the benefits and reimbursement of such practices.

## Care Coordination and Pricing Failures: The Future of the Hospital

Demand for acute care, especially ICU, has increased drastically.^10^ Since these costly functions are often cross-subsidized from more chronic and elective care – now delivered way beyond their normal utilization levels – hospitals will come under financial stress. In the current situation, elective care should be detached from acute hospital care to reduce risk of COVID-19 infections. Christensen et al have famously expressed the need to redesign the care delivery system.^11^ The efficiency of hospitals could structurally be augmented by clearer demarcations between unstandardized, standardized, and network-related processes. However, up to now, we have not witnessed a revolution along those lines. COVID-19 presents the external shock to spur disruptive innovation as suggested by Christensen et al.^11^

In both acute and tertiary care, stronger coordination seems necessary to optimize utilization and to reduce overhead costs.^12^ The more so, since COVID-19 illustrates the need for rapid scalability of these vital functions in the event of a health-related emergency. For example, a Dutch national coordination center for allocating patients in need of acute care has alleviated pressure on the ICU in regions struck hardest by the virus. Effective coordination limits costs of excess capacity of expensive infrastructure such as ICU.^13^ Building spare capacity and keeping workers that have left to other positions, aligned to the health system in urgent cases may be other efficient strategies. A more centralized coordination of certain care services has more possibilities to realize this expeditiously. However, this crisis also shows that there exists substantial difference between countries on such matters.

In elective care, coordination can be less strict. Separating acute and elective care also makes it more easier for the latter to continue its service, if the former is under threat. In all cases the high mortality of COVID-19 patients with underlying comorbidities, illustrates the importance to fundamentally redesign the delivery system towards multimorbidity, for example seamless care in networks. In the future, hospitals might create separate hub functions that deliver the knowledge- and technical infrastructure to integrate care (for patients with co-morbidities). The current pricing system should steer away from the cross-subsidies that prevent such changes. It also has a role to play in preventing administrative complexity, by putting a price tag on it: treatment reimbursement that is tied to high-cost but low-value administrative procedures should be made more expensive to the payers.

Timely and integrated data are not only key in the fight against COVID-19. Better information systems also act as vehicles to provide accurate and timely information that can widely be used to improve health across providers and all other stakeholders in the system. Recently, South-Korea seems to be the spearhead that shows the value of readily available and integrated data. Others should follow their steps.

## Not Wasting This Opportunity: Policy Windows Are Open Temporarily

In our politicizing world, one remarkable aspect of the current situation, is the sudden reliance on experts and facts. One by one politicians suddenly realize the importance of evidence-based policy-making, keeping alternative facts at bay. Competition and crisis containment seem to be an uneasy marriage. Fact driven professionalism as a decent way of governing what needs to be done made a comeback. It might be the best way to steer COVID-19. Healthcare professionals come to the fore as strong policy entrepreneurs as well. However, these experts and professionals should not shy away from some other responsibilities to strengthen the delivery systems where they work. Besides all its negative consequences, COVID-19 has opened windows of opportunity where the streams of policy solutions, politics and problems, can come together, thus can be used for the good of our healthcare systems. In this perspective we have argued that current events can canalize some very needy reforms to make our systems more sustainable. Let us not waste the moment, otherwise it will pass and the vested interests will come back to pursue their own agendas.

## Acknowledgements

We like to thank Gert Westert for his contribution to this paper.

## Ethical issues

Not applicable.

## Competing interests

Authors declare that they have no competing interests.

## Authors’ contributions

All authors contributed to the idea of work and all have co-written the manuscript. All authors approved the final version of the manuscript.
